# Innovations in Clinical Research Design and Conduct in Psychiatry: Shifting to Pragmatic Approaches

**DOI:** 10.4306/pi.2009.6.1.1

**Published:** 2009-03-31

**Authors:** David M. Marks, Thanaseelan J, Chi-Un Pae

**Affiliations:** 1 Department of Psychiatry and Behavioral Sciences, Duke University, Durham, NC, USA.; 2 Department of Psychiatry, The Catholic University of Korea College of Medicine, Seoul, Korea.

**Keywords:** Innovation, Strategies, Psychiatric research

## Abstract

The classically structured clinical trial does not offer enough flexibility to make use of continuously emerging knowledge that is generated as the trial progresses. In this regard, there are consistent issues impeding effective psychiatric research, including limitations in efficiency, difficulty demonstrating significant differences between treatment arms, poor external validity, and ethical constraints. For example, research in the field of psychiatry shows that it is growing increasingly more challenging to demonstrate superiority of interventions to placebo in part related to the increasing placebo response rates. Various design innovations and other tricks of the trade have surfaced to improve sensitivity towards detecting drug-placebo differences and reduce sources of bias in psychiatric research. Diverse strategies have been developed to address these obstacles and improve the outcomes of clinical research in psychiatry. The current review highlights many of these innovations and describes examples of their practical use, mainly focusing on the study design and conduct perspectives. In the study design issues, adaptive, equipoise stratified, sequential parallel and effectiveness design will be explored. The proper strategies for pragmatic and ethical conduction of clinical trials will be also discussed in-depth.

## Introduction

The scope of available interventions in the treatment of psychiatric disorders has grown in recent decades in part through the diligent work of researchers investigating the usefulness of such interventions. Psychiatric research has become increasingly innovative and sophisticated in order to optimize ethical conduct, maximize patient safety, and increase the ability of potentially effective treatments to demonstrate superiority to placebo or noninferiority to standard of care interventions. Due to mounting concerns that potentially effective pharmacotherapies are failing to outperform placebo in clinical trials, strategies are evolving to increase efficiency, sensitivity, and statistical power and to reduce bias.

Additionally, emphasis is now being placed on the generalizability and external validity of psychiatric research, since patients encountered in clinical practice often do not mirror populations of patients enrolled in industry-sponsored clinical trials. This emphasis has spawned large effectiveness trials designed to inform clinicians about the relative strengths of already marketed treatments in psychiatry. Innovations have also been made in the ethical design and conduct of psychiatric research by allowing greater flexibility in how patients are randomized to treatment.

Modern study designs are adaptable to specific subject characteristics and to early data collected over the course of the trial, and these innovations serve to expose patients to treatments that are more likely to be useful. The manner in which informed consent is collected is continuing to evolve in innovative ways, and this is particularly important given the vulnerable nature of psychiatric patients. The current review introduces various innovations that have been employed in clinical research design and conduct and describes their intended benefits.

## Study Design Innovations

### Adaptive designs

Adaptive design can be defined as a clinical study design that uses accumulating data to decide how to modify aspects of the study as it continues without undermining the validity and integrity of the trial.^1^ In the opinion of some authors, adaptive design use in clinical research refers to modifications that are incorporated into the study protocol and not developed on an ad-hoc basis to remedy inadequate planning.^1^ An example of adaptive design meeting this definition includes running patients through multiple interventions (i.e. treatment algorithms) based on their response to such interventions. These strategies individualize treatment via decision rules that recommend when and for whom the treatment should change.^2^

On the other hand, it has been suggested that adaptive designs include any adaptations or modifications made to the study or statistical procedures during the conduct of the trial, including those determined in amendments to clinical trial protocols.^3^ Multiple examples of such adaptations have been described, including but not limited to 1) sample Size Reestimation--adjustment of sample size to accommodate inaccurate prediction of effect size or variance (thus preserving statistical power),^1^ 2) adaptive Treatment Allocation (and Adaptive Dose-Finding)-alteration of the ratio of allocation to treatment arms based on ethical grounds when an interim analysis reveals marked differences in effectiveness or tolerability between arms,^1^,^4^ 3) adaptive Hypothesis-change in the primary hypothesis of the study, which may include change in the primary outcome measure, shift from superiority hypothesis to noninferiority hypothesis, interchanging the null and alternative hypotheses, and various other modifications of elements of the study hypothesis.

Adaptive designs (whether a priori or ad hoc) offer the ethical advantage of better treatment of participants, the strategic advantage of improving probability of study success, and the potential economic advantage of sparing resources by reducing participant numbers. Yet, adaptive designs run the risk of increasing the chance of type I error, although authors have described multiple methods to combat this risk.^3^

There are inherent logistical issues in the use of some adaptive strategies, since data must be collected and analyzed rapidly and modifications resulting from this analysis must be quickly devised and approved by regulatory agencies. Overall, regulatory agencies have been slow to accept the application of adaptive designs in clinical research. A PhRMA Working Group was formed in 2005 to evaluate the benefits and challenges of adaptive designs with the goal of increasing usage and regulatory acceptance of adaptive clinical trial designs.^1^

### Equipoise stratified design

Traditional randomized clinical trials are conducted in such a way that the treatment preferences of participants and investigators are ignored; randomization into two or more treatment arms occurs without the influence of clinician or patient preference.

This design seems to be the most scientifically pure, but in multiple arm clinical trials in human subjects it can unnaturally subject patients to treatments they do not prefer and restrict clinical investigators from exercising judgment to offer treatments they believe to be superior. Lavori et al.^5^ have developed a clinical trial design called the equipoise stratified design which allows some flexibility in the randomization process by allowing participants and investigators to choose among groups ("e-stratum") containing multiple study arms. The clinician's and patient's preferences are identified in advance of assignment to treatment, so that the random assignment can be made within strata defined by those preferences. For example, in the recent Sequenced Treatment Alternatives to Relieve Depression (STAR*D) study,^6^ patients who failed to respond adequately to the antidepressant citalopram could choose to be assigned to one of two groups of interventions, "switch" or "augment". Patients opting for the "switch" stratum were randomized equally to four arms consisting of three different antidepressants and cognitive therapy. Patients opting for the "augment" stratum remained on citalopram and were randomized equally to three arms consisting of two different adjunctive agents and adjunctive cognitive therapy. This model has an element of adaptive design in that preference for the "augment" stratum may have been preferentially chosen if patients had a partial response to citalopram.

This issue illustrates the ethical advantage of the equipoise stratified design in that it allows individual patient experience to be considered prior to randomization, and this flexibility theoretically enhances generalizability and efficiency of enrollment. The equipoise stratified design is applicable only when treatment arms can be grouped into strata, which limits its use to a subset of clinical research studies.

### Sequential parallel design

High placebo response rates have limited the ability of psychiatric clinical trials to detect efficacy with pharmacological interventions that ultimately have gained acceptance as effective treatments. Even when such interventions lead to measured improvement, they risk nonseparation from placebo due to measured improvement in the placebo arms of clinical trials.

Many of the research interventions discussed in the current review are aimed at combating the trend of high placebo response rates. Included in these interventions is the sequential parallel design, a new study design proposed by Fava et al.^7^ aimed at increasing the efficacy of double blind placebo controlled psychiatric clinical trials by reducing placebo response rates and required sample size. The sequential parallel design combines data from a standard parallel placebo-controlled trial with data from a second phase in which patients in the placebo arm who fail to improve are again randomized to study drug or placebo. Although such a design naturally extends the length of a clinical trial, it has been asserted that in antidepressant trials the duration of each phase can be shorter than a traditional parallel group trial. Other authors have expanded upon this design concept. The original design by Fava et al. proposed to allow responders in the first phase to continue on the drug in an open-label fashion during the second phase.^7^ Tamura et al.^8^ proposed modifications to the sequential parallel design, including retaining patients on blinded medication throughout both phases of the trial with investigators blinded to the criteria for response and the exact timing of the initiation of the second phase.^8^ These authors also propose to keep first phase study drug nonresponders on the drug during the second phase in order to obtain additional tolerability data^8^ in contrast to the suggestion by Fava et al. to switch study drug nonresponders to placebo during the second phase.^7^

Statistical modeling has demonstrated that compared to traditional parallel design, the sequential parallel design allows reduction in sample size of 20-25% while retaining similar power.^8^ This reduction leads to financial savings despite longer trial duration,^8^ and we suspect that the reduced sample size would shorten total study length in most cases by reducing the enrollment period.

### Effectiveness studies

Industry-sponsored registration trials in psychiatry tend to follow a standard design which involves short-term, double-blind, placebo-controlled, parallel group study of patients with acute episodes or exacerbations of chronic illness. Patients are excluded if they have substantial medical comorbidity, and usually concomitant medication treatments are limited. Primary endpoints are typically investigator-scored efficacy measures (rating scales), and these trials are sometimes termed "efficacy studies". Such research has been criticized in lacking external validity and it has been asserted that these efficacy studies do not provide sufficient information to clinicians in real-world settings.^9^

In contrast, some studies have been conducted with the intent of providing clinicians with useful practical data regarding the comparative effectiveness of marketed medications. In particular, the Clinical Antipsychotic Trials of Intervention Effectiveness (CATIE) was aimed at detecting differences in effectiveness and tolerability among available antipsychotic drugs.^10^ CATIE compared several antipsychotics in a large sample of schizophrenic patients with relatively few exclusion criteria, and the primary outcome measure of "effectiveness" was "all cause discontinuation" in accordance with the desire to study a clinically-relevant and practical marker of efficacy and tolerability. Reasons for treatment discontinuation were compared such that clinicians now have a better understanding of how the various agents compare on efficacy, side effects, and overall treatment effectiveness in a community population of schizophrenics. Effectiveness studies are large, simple trials focusing on a small number of easily-measured endpoints and are therefore able to enroll large numbers of participants to answer "real-world" clinical questions regarding the effectiveness of treatments.^9^ These studies aim to enroll typical community patients by having relatively lenient inclusion/exclusion criteria and concomitant medication restrictions, thus maximizing external validity.

Table 1 summarizes the differences between traditional randomized, double-blind placebo-controlled clinical trials (efficacy trials) and effectiveness trials.

## Study Conduct Innovations: Reducing Placebo Response Rates

It is widely accepted that research on some psychiatric disorders is prone to high placebo response rates. Psychotic disorders and obsessive compulsive disorder (OCD) tend to have low placebo response rates in contrast to the high rates seen in studies of major depressive disorder (MDD), generalized anxiety disorder (GAD), and panic disorder.^11^ Data indicate that placebo response rates in antidepressant trials exceed 30%,^11^,^12^ and more than half of the clinical trials for approved antidepressants failed to show drug-placebo separation.^11^,^12^

The reasons for placebo response in psychiatric trials are numerous and beyond the scope of this review, but some of the main contributions to placebo response relate to interactions between research site staff and subjects as well as rating scale bias. Rating scale bias occurs when investigators deliberately or unconsciously inflate the scores of efficacy measures at screen or randomization to include subjects that might otherwise fall just below the cutoff for inclusion in the trial. If scores are inflated to qualify marginal patients who enter the placebo arm, subsequent ratings will presumably show more accurate ratings leading to false impression of improvement. Some innovative ways of addressing this tendency have been successfully implemented, including unlinking the inclusion criteria and the primary efficacy measure, and blinding the subjects and investigators to the time of randomization. For example, the two registration trials leading to the United States Food and Drug Administration (US FDA) approval of quetiapine for the treatment of bipolar depression^13^,^14^ and the two registration trials leading the US FDA approval of aripiprazole as adjunctive therapy in MDD^15^,^16^ used the Montgomery Asberg Depression Rating Scale (MADRS) as the primary efficacy measure while using the Hamilton Rating Scale for Depression (HAM-D) to qualify patients at study screen and at randomization. This strategy was designed so that inflation of rating scale scores (HAM-D) in order to enroll subjects at screen and randomization would not lead to exaggerated scores on the primary efficacy measure (MADRS). Furthermore, some studies have adopted the design of blinded time or randomization to combat subject expectations of improvement and the tendency of raters to inflate scores at the randomization visit. The aripiprazole registration trials referenced above blinded patients but not investigators to the time of randomization.^15^,^16^

Although most sponsors orient investigative site staff and provide good clinical practice training to the investigators at the beginning of every multi-center trial, the heterogeneity in the management of patients' expectation among centers, and the occurrence of diagnostic misclassification remains relevant.^17^ In some centers, the interaction between the investigators and the patients may favor the occurrence of strong placebo responses, affecting most of the subjects recruited at the center, and reducing the possibility of detecting any treatment effects.^18^

Some authors have gone so far as developing a method to determine which investigative sites yield high placebo response in a particular study and proposing that data from underperforming sites be dropped.^19^ This premise is based on the notion that sites differ in their placebo response rates, possibly as a function of how sites manage patient expectations. The determination of individual sites' placebo response rates has been done retrospectively based on a signal detection approach,^20^ and authors have proposed a way to prospectively measure the response rates of additional patients treated in a double blind fashion with placebo outside the randomization sequence ("calibrator signal").

Table 2 summarizes the issues related to high placebo response in clinical trials.

## Innovations in Informed Consent

Informed consent may be construed as one of the most fundamental ethical principles in human subjects research. Investigators bear a responsibility to ensure that subjects are able to voluntarily agree to research participation, and one of the primary roles of internal review boards (IRBs) is to ensure appropriate informed consent procedures. Yet, the leading cause of US FDA warning letters and 483 letters to investigators is deficiency in informed consent, and research has demonstrated frequent misunderstanding by subjects in clinical research despite elaborate informed consent processes.^21^,^22^

The informed consent process has been conceptually divided into multiple steps which include 1) assessing the decision-making capacity or competence of the prospective research volunteer, 2) disclosing relevant information about the proposed research, 3) ensuring that the prospective volunteer understands the information, 4) ensuring that the prospective volunteer be positioned to make a voluntary choice, and 5) authorizing a decision by the prospective volunteer and, if affirmative, having him or her sign a consent form.^23^,^24^ Various authors have suggested that informed consent information be presented in multimedia format such as Powerpoint^25^ or CD-ROM,^24^ although it has been noted that more elaborate presentations might unduly persuade potential subjects to participate.^24^

In addition, questionnaires have successfully been administered to improve the assessment of potential subjects' understanding of what is involved in specific research protocols,^26^ and a unique study improved the informed consent dialogue by embedding queries about the study process directly into the informed consent document.^25^ Such measures allow the investigator to more formally assess potential subjects' understanding of the implications of research participation. Schwartz and Applebaum have advocated that research staff record informed consent discussions for the purpose of quality control audit by the investigators and by regulatory agencies such as IRBs.^22^

## Discussion

Just as the science of medicine continuously evolves to provide better treatments, methodology evolves to enhance the sensitivity, efficiency, and moral integrity of clinical research. This evolution has various aims, which include improving the ability to determine whether specific interventions are of benefit, reducing the cost associated with research, improving external validity, maximizing subject and clinician choice while preserving data integrity, exposing a greater number of subjects to the most effective and tolerable treatments, and safeguarding the safety and welfare of research subjects.

Research in the field of psychiatry shows that it is growing increasingly more challenging to demonstrate superiority of interventions to placebo in part related to the increasing placebo response rates.^27^ Various design innovations and other tricks of the trade have surfaced to improve sensitivity towards detecting drug-placebo differences and reduce sources of bias in psychiatric research. Ultimately, it is an empirical question whether these many innovations successfully improve efficiency and ability to detect differences in efficacy between various interventions or between interventions and placebo.

Additionally, design innovations have enhanced the ethical conduct of research in several ways. Certain adaptive designs permit the use of treatment algorithms to tailor treatments to individual subject responses during a study, while other adaptive designs allow modifications to be made in protocol conduct based on early data collected over the course of the study in such a way that more patients are exposed to treatments demonstrating safety and efficacy. Innovations in research ethics also include improvements in the way informed consent is acquired and monitored. This is particularly relevant in psychiatry, since many patients with mental illness may have reduced capacity to understand the risks, benefits, and alternatives related to study participation. It seems intuitive that the various innovations discussed are successful at improving the ethical conduct of psychiatric research, although in some ways this is an empirical question as well.

## Figures and Tables

**TABLE 1:**
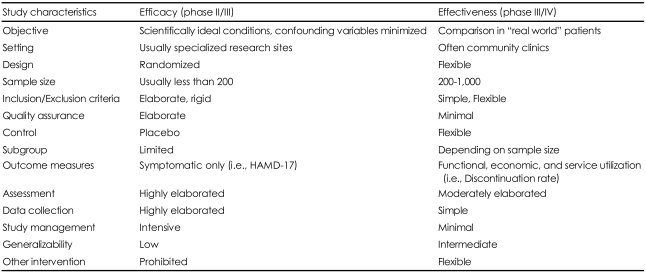
A comparison between efficacy and effectiveness trials

HAMD: Hamilton Rating Scale for Depression

**TABLE 2:**
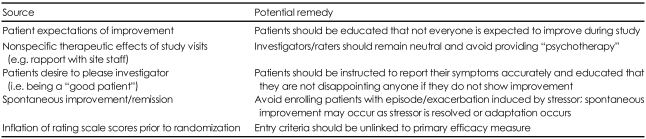
Issues contributing to placebo response in psychiatric clinical trials
